# Sponges and Their Microbiomes Show Similar Community Metrics Across Impacted and Well-Preserved Reefs

**DOI:** 10.3389/fmicb.2019.01961

**Published:** 2019-08-22

**Authors:** Marta Turon, Joan Cáliz, Xavier Triadó-Margarit, Emilio O. Casamayor, Maria J. Uriz

**Affiliations:** Centre d’Estudis Avançats de Blanes, CEAB-CSIC, Girona, Spain

**Keywords:** ecology, sponges, microbiomes, diversity, resilience, contrasting environments, eutrophication

## Abstract

Sponge diversity has been reported to decrease from well-preserved to polluted environments, but whether diversity and intra-species variation of their associated microbiomes also change as function of environmental quality remains unknown. Our study aimed to assess whether microbiome composition and structure are related to the proliferation of some sponges and not others under degraded conditions. We characterized the most frequent sponges and their associated bacteria in two close areas (impacted and well-preserved) of Nha Trang Bay (Indo-Pacific). Sponge assemblages were richer and more diverse in the well-preserved reefs, but more abundant (individuals/m. transect) in the impacted environments, where two species (*Clathria reinwardti* and *Amphimedon paraviridis*) dominated. Sponge microbiomes from the polluted zones had, in general, lower bacterial diversity and core size and consequently, higher intra-species dispersion than microbiomes of sponges from the well-preserved environments. Microbial communities reflect the reduction of diversity and richness shown by their host sponges. In this sense, sponges with less complex and more variable microbiomes proliferate under degraded environmental conditions, following the ecological paradigm that negatively correlates community diversity and environmental degradation. Thereby, the diversity and structure of sponge microbiomes might indirectly determine the presence and proliferation of sponge species in certain habitats.

## Introduction

Sponges are key invertebrates in marine benthic ecosystems where they play essential functions in many ecological processes. Besides increasing benthic diversity by supplying ecological niches to other organisms, they contribute to benthic–pelagic coupling by exchanging particulate and dissolved organic matter with the water column (Richter et al., 2001; Morganti et al., 2017). Sponges also participate in marine biogeochemical fluxes and some species may also show detoxifying potential by transforming noxious products present in polluted waters through the interplay of symbiotic bacteria (Loredana et al., 2017).

Microbes have been intimate partners of sponges since the Pre-Cambrian (Wilkinson, 1984). They can represent up to ca. 50% in volume of the microbial-sponge holobiont in some sponge species (Uriz et al., 2012) and are taxonomically and metabolically diverse in most cases (Weisz et al., 2007; Thomas et al., 2016). Thus, it is hard to envisage the causes of sponge success or failure without considering its accompanying bacterial microbiome. Indeed, unbalancing of their microbial symbioses has been considered to trigger extensive mass mortalities of sponges in the Mediterranean (Webster et al., 2008; Cebrian et al., 2011), and Red Sea (Gao et al., 2014). In addition, some purported benefits that sponges may obtain from their associations with microbes have also been proposed such as, antifungal activity, production of bioactive compounds against predation, roles in nitrogen and carbon cycle and vitamin biosynthesis (Schmidt et al., 2000; Freeman and Thacker, 2011; Hentschel et al., 2012; Freeman et al., 2013), but rarely have been experimentally demonstrated (de Voogd et al., 2015; Garate et al., 2015).

In contrast to the spatial and temporal variations reported for microbial communities in seawater (Zeglin, 2015; Glasl et al., 2017), the structure of the sponge microbiome does not vary substantially in the same species along geographical and bathymetrical ranges or over temperature, eutrophication or irradiance shifts (Hentschel et al., 2002; Erwin et al., 2012; Pita et al., 2013a,b; Luter et al., 2014; Strand et al., 2017). Although several experimental studies reported microbiome shifts under strong environmental changes in some host species (Mohamed et al., 2008; Fan et al., 2013; Lesser et al., 2016; Webster et al., 2016; Pineda et al., 2017; Weigel and Erwin, 2017; Glasl et al., 2018; Ramsby et al., 2018) they only inform us about short term changes, which might reflect temporal responses to environmental stresses.

Sponges usually show a high species diversity in well-preserved ecosystems (Van Soest et al., 2012), which has been suggested to decrease under anthropogenic pressures (Easson et al., 2015). Shifts in nutrient cycling and ecosystem functioning, which occur in degraded reef systems (Bell et al., 2013, 2018; Easson et al., 2015), are considered to be responsible for decreases in sponge biodiversity. However, a few, likely opportunistic, sponge species have been reported to inhabit and even dominate degraded coral reefs (Maliao et al., 2008). How these sponges cope with the potentially noxious products of polluted waters and whether particular sponge-associated bacteria are involved or not in their ecological success are challenging issues poorly understood (Douglas, 2014). Indeed, little is known about the role (if any) that symbiotic bacteria may play in shaping the ecological distribution of sponge species and whether the decrease of sponge diversity observed in perturbed assemblages also involves a lower diversity of their associated bacterial communities.

In this study, we explored whether microbiomes of sponges inhabiting degraded environments show differential characteristics, in such a way that they might influence the sponge distribution and proliferation in those adverse conditions. With this goal, we characterized the most frequent sponges and their associated bacteria in two close areas of Nha Trang Bay (Indo-Pacific) subjected to contrasting environmental conditions: well-preserved and impacted coral systems. In particular, we looked for sponge-associated bacteria in the few sponge species able to proliferate in the polluted zones.

Nha Trang bay is located in central Vietnam and harbors one of the highest coral diversities in the area (Latypov, 2011). Unfortunately, the health of the local ecosystems is being threatened by an increase of human activities leading to an alarming degradation of the bay in some zones (Latypov, 2015). The islands located closer to the city and the port of Nha Trang show a higher degree of anthropogenic impact, with higher sedimentation fluxes and lower water transparency compared to the ones located farther from the coast line (Latypov, 2006; Tkachenko et al., 2016). Moreover, Vietnam has been ranked the world’s third largest producer of farmed food (Nguyen et al., 2016) and certain areas of Nha Trang bay are highly impacted by mariculture activities, which also produce eutrophication and release of xenobiotics to the water (Nguyen et al., 2016). Tkachenko et al. (2016) reported the concentration of nutrients in areas close to our sampling sites. The nutrient values were 2.63 μM ± 0.21 for dissolved inorganic nitrogen and 0.29 μM ± 0.11 for phosphorus at the impacted sites (culture cages) and 2.41 μM ± 0.2 and 0.28 μM ± 0.08 at the unperturbed sites. As a result, the native *Acropora* coral assemblages have been replaced by the more resistant to silting *Millepora* communities in these perturbed areas (Latypov, 2015; Tkachenko et al., 2016). Conversely, several outer areas of Nha Trang Bay, such as Hun Mun, receive a low anthropogenic impact and still present well-developed *Acropora* communities with high coral coverage (ca. 60–70%) (Tkachenko et al., 2016).

## Materials and Methods

### Sample Collection and DNA Extraction

Quantitative sponge sampling was approached by SCUBA diving by randomly placing a total of 13, 25 m-long transect lines between 3–9 m depth along both well-preserved and impacted areas of Nha Trang Bay (Supplementary Figure S1). This quantitative method has been traditionally used for biodiversity studies in coral reefs and other structurally complex habitats (Loya, 1978) and provides a good approach on the abundance (density or coverage) of the non-cryptic fraction of the reef benthos. Specimens that were crossed by the metric tape along the line transect were sampled. The well-preserved areas were coral reefs and rocky shores of the eastern part of Hun Mun Island and the southern part of Hun Tre Island, considered to be the most well preserved areas of the bay (Tkachenko et al., 2016). The impacted targeted zones were 2 km apart from the well-preserved areas, next to Dambay region, which harbors an intensive mariculture system (lobster caging) that causes chronic eutrophication in the area (Tkachenko et al., 2016). Overall, the quantitative sampling provided 203 sponge samples, from which 71 individuals were from the impacted areas and 132 individuals from the well-preserved environments.

Each sponge individual fitting within a line transect was photographed and a piece of ca. 3 cm^2^ (whenever possible) was collected in a 50 mL Falcon tube with seawater. Seawater was immediately replaced by 100% ethanol once on board. Back in the lab, the ethanol was replaced twice with fresh absolute ethanol again for a good sample preservation. DNA was extracted following the protocol of DNeasy Blood & Tissue Kit (Qiagen). Triplicate plankton samples were taken from the three sampling locations where the ecological transects were performed (i.e., Dambay, Hun Mun and Nock Island). Two liters of water were collected and sequentially filtered throughout 5-μm, to remove undesired plankton components, and then throughout 0.22 μm polycarbonate membranes. The size fraction between 5 and 0.22 μm was processed for DNA extraction. Membranes were enzymatically digested with lysozyme, proteinase K and sodium dodecylsulfate and afterward, DNA was extracted with phenol:chloroform-isoamyl alcohol (25:24:1, vol/vol/vol) and chloroform:isoamyl alcohol (24:1, vol/vol).

Sponge species were previously identified in Turon et al. (2018) by morphological features (Hooper and Van Soest, 2002) and molecular markers. Preparation of spicules and histological sections were made from specimens’ subsamples and observed under both light and scanning electron microscopes.

### 16S rRNA Gene Sequencing and Processing

Only representative sponge species (*n* = 18) at each environment, which appeared replicated in the quantitative sampling, were used for the study of microbial symbionts. Replicates varied from 2 to 9, depending on the species abundance (n. individuals) and material availability (sponge fragment size), as the sampling was intended to reflect the sponge diversity and abundance at each environment (Table 1). PCR and high-throughput multiplexed 16S rRNA gene amplicon Illumina MiSeq sequencing, were carried out following the genomic core facilities and methods of the MrDNA Lab (Texas, United States)^1^. The variable V4 region of the bacterial 16S rRNA gene was amplified using the primers 564F (5′AYTGGGYDTAAAGNG-3′) and 785R (5′TACNVGGGTATCTAATCC-3′) (c.a. 250 nt) (Klindworth et al., 2013). Raw rRNA gene sequences were processed separately using the UPARSE pipeline (Edgar, 2017). A quality check was applied to our dataset with the fastq_filter command and the arguments –fastq_trunclen 208 –fastq_maxee 0.25. Sequences were then dereplicated with the – derep_fulllength command and sorted by size (-sortbysize command) in Usearch 9.2 version. Denoising (error-correction) of amplicons was performed following the UNOISE pipeline (Edgar, 2016) using the - unosie2 command. This algorithm removed chimeras, reads with sequencing errors, PhiX, and low complexity sequences due to Illumina artifacts, and generates ZOTUs (“zero-radius” OTUs) with 100% identity sequences. Finally, -usearch_global command with identity threshold set at 0.97 was applied to our dataset. Taxonomic assignment was done with SINA v1.2.11 (Pruesse et al., 2012) using SILVA 128 database. Sequences with low identity (< 75%) and sequences identified as mitochondria or chloroplasts were removed from the analysis. In order to minimize biased effects for differences in sampling effort, the original bacterial ZOTU table was rarefied at a minimum reads threshold of 41000, using QIIME (Caporaso et al., 2010).

**TABLE 1:** Species replicates used for the microbiome study.

**Species**	**N. Impacted Env.**	**N. W-P Env.**
*Aaptos suberitoides*	0	7
*Amphimedon paraviridis*	5	0
*Antho* (*Antho*) sp.	0	3
*Callyspongia* sp.	0	2
*Clathria reinwardti*	9	0
*Clathria (Isociella) skia*	4	0
*Dendroxea* sp.	0	2
*Dysidea* sp.	0	3
*Amphimedon sulcata*	3	6
*Haliclona* (*Reniera*) sp.	0	3
*Monanchora unguiculata*	0	3
*Mycale* (*Arenochalina*) sp.	2	1
*Neofibularia* sp.	0	4
*Phorbas* sp.	0	3
*Protosuberites proteus*	0	2
*Suberea fusca*	0	3
*Thrinacophora cervicornis*	0	2

### Sponge and Bacterial Community Analysis

Statistical analyses were run in the R environment (R Core Team, 2013). Community ecology related parameters were calculated using the vegan v2.5-1 (Oksanen et al., 2017) and iNEXT v2.0.15 packages (Hsieh et al., 2018), and figures were drawn with ggplot2 3.0.0 (Wickham, 2009).

We determined sponge species composition on impacted and well-preserved environments: the number of sponge individuals was standardized per meter of sampled transect to minimize the bias of the sampling effort in the two habitats. Shannon diversity indices and species richness were calculated for both environments using the *ChaoEntropy* and *ChaoSpecies* functions, respectively, and integrated curves that smoothly link rarefaction (interpolation) and prediction (extrapolation) were computed for both variables (Chao et al., 2014) to facilitate their comparison of between habitats (Supplementary Figure S2). Additionally, we used the specaccum function with 1000 permutation of the vegan package to represent the accumulated number of species per sampled meter in each habitat (Supplementary Figure S3).

A Venn diagram of total species richness in both environments was generated using the *eulerr* package (Larson et al., 2018) and *t-*tests were conducted to assess differences in community metrics between sites. We calculated the Bray–Curtis dissimilarity of species composition of all transects to assess the beta diversity patterns between environments.

We performed a hierarchical cluster analysis (Ward method) based on Bray–Curtis dissimilarity matrix using the rarefied ZOTU table to determine whether sponge bacterial communities were more similar among replicates from the same species than between impacted and well-preserved environments. To test the effects of host identity and environment on structuring the sponge bacterial communities, we used PERMANOVA (Anderson, 2001) based on 999 permutations as implemented in *adonis* function. A heatmap was generated for the most abundant bacterial families (>1% relative abundance in any of the samples). We performed and Indicator Value (IndVal) (Dufrêne and Legendre, 1997) analysis to detect if particular microbial taxa were differentially found at each site using the *labdsv* package (Roberts and Roberts, 2016) in R. We set the Indval threshold to 0.7 and the *p*-value for significance at 0.01.

We compared three main bacterial community features between the sponge assemblages from impacted and well-preserved environments: (i) intra-species dispersion (beta diversity), (ii) Shannon diversity and (iii) core size (explained below). For these comparisons, we included *Amphimedon sulcata* and *Mycale* (*Arenochalina*) sp., for which we had replicates in the impacted and well-preserved environments, within the category of sponges able to survive in impacted sites.

Only two species inhabited both environments [*A. sulcata* and *Mycale (Arenochalina)* sp.]. However, only one individual of *Mycale* (*Arenochalina*) sp. was found in the well-preserved habitats, preventing statistically based comparisons between environments for this species. *Betadisper* and *Shannon* functions were used to calculate beta and alpha diversity measures, respectively. We defined the bacterial core of each sponge species considering the ZOTUs present across all species replicates. The core size of each individual represented the percentage of core bacteria (of the sponge species) with respect to the total bacteria present in that individual. *T-*test and *Kruskal–Wallis* test were used to detect significant differences between sponge species from impacted and well-preserved environments. *Pearson* correlation was used to detect the relationship between intra-species dispersion and bacterial core size for each sponge species.

## Results

### Sponge Assemblages in Two Contrasting Environments

**FIGURE 1:**
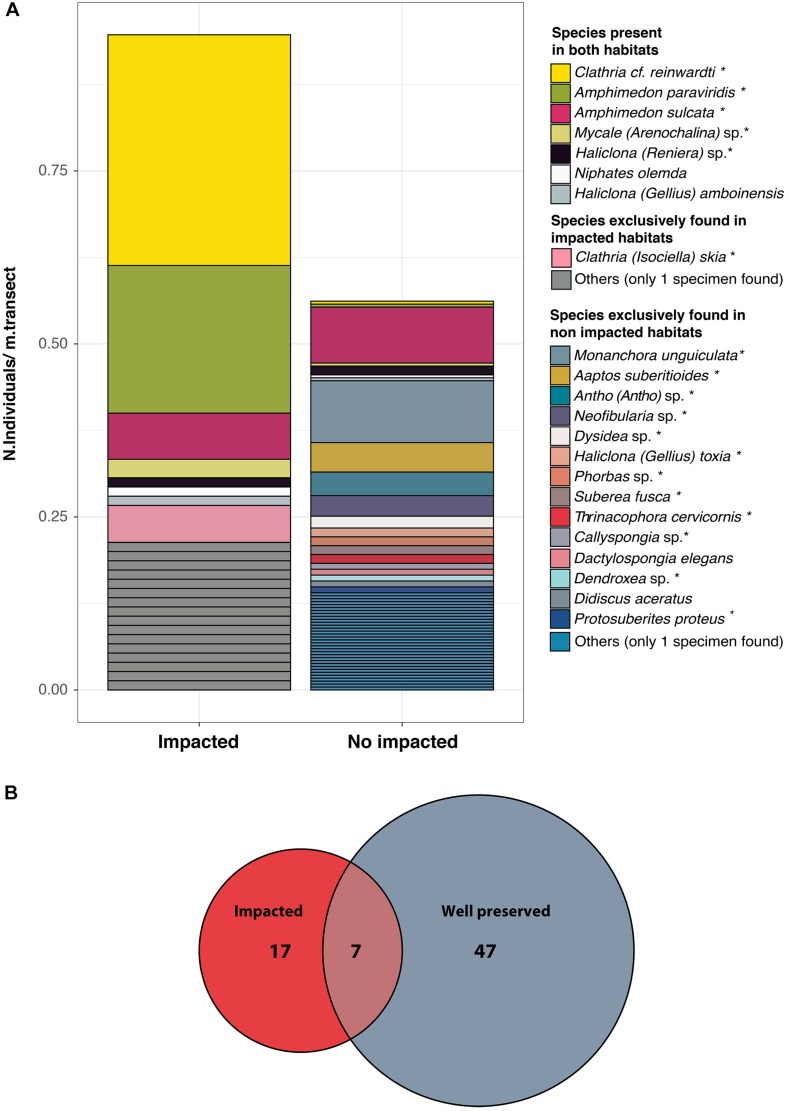
**(A)** Bar plots of sponge species composition and abundance at the well-preserved and impacted habitats. *Y*-axis represents the numbers of individuals per meter of transect. **(B)** Venn diagram of total species richness and the species overlap between well-preserved (gray) and impacted (red) environments. ^∗^ Indicates species for which microbiome has been analyzed.

A total of 71 sponge species were identified in the overall sampling area (Figure 1). The impacted and well-preserved environments showed strong differences in terms of sponge richness, diversity, and density (Figure 1 and Supplementary Figure S2). Sponge richness and diversity were much lower in the impacted sites than in the well-preserved environments (*t*-test: *p*-value = 0.039, *p*-value = 0.037, respectively), although the respective rarefaction curves per number of individuals sampled did not reach the saturation point, in particular for species richness (Supplementary Figure S2). The rarefaction curves of accumulated species per sampled area neither reached the saturation point (Supplementary Figure S3).

**FIGURE 2:**
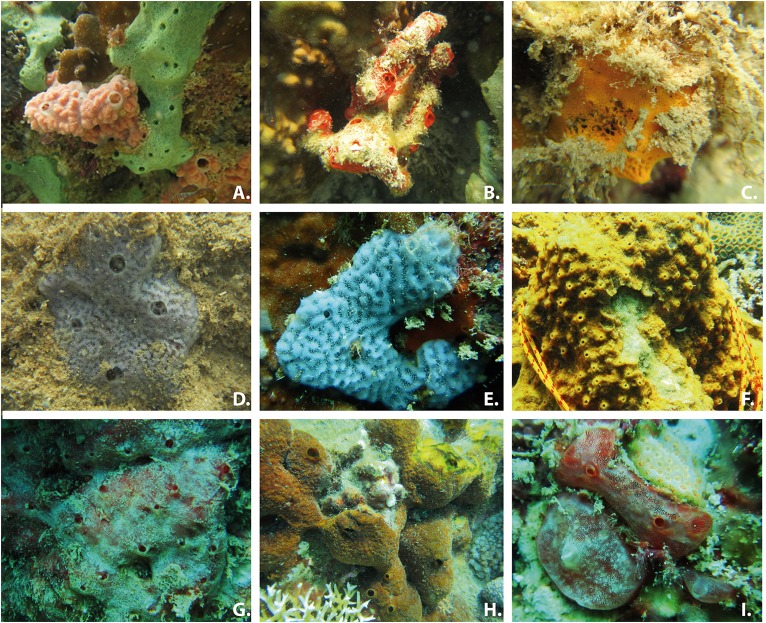
Pictures of the most common sponges of the impacted **(A–D)** and the well-preserved **(E–I)** habitats: **(A)** *Amphimedon paraviridis* (greenish) and *Clathria reinwardti* (pinkish), **(B)** *Clathria* (*Isociella*) *skia*, **(C)** *Mycale* (*Arenochalina*) sp., **(D)** *Amphimedon sulcata* (in the impacted environment), **(E)** *A. sulcata* (in the well-preserved environment), **(F)** *Neofibularia* sp., **(G)** *Antho* (*Antho*) sp., **(H)** *Aaptos suberitioides* and **(I)** *Monanchora unguiculata.*

Conversely, the overall sponge density was higher (*t*-test: *p*-value = 0.05) in the impacted (0.94 individuals/m) than in the well-preserved environments (0.56 individuals/m). Most species were environment-specific with only seven species found in both environments, though all of them but *A. sulcata* were much more abundant at the polluted sites (Figure 1). The well-preserved habitats harbored a more diverse and evenly distributed sponge assemblage with *Monanchora unguiculata, A. sulcata, Antho (Antho)* sp.,* Aaptos suberitoides*, and *Neofibularia* sp. (Figures 1, 2) being the most abundant species. Conversely, the impacted habitats showed a community mostly dominated by *Clathria reinwardti* and *A. paraviridis* (Figures 1, 2) (>50% of the specimens sampled). Moreover, significant differences in beta diversity for species composition were found between sites (adonis, *F* = 2.72, *df* = 1, *R^2^* = 0.19, *P* = 0.01).

### Sponge-Associated Bacterial Communities

**FIGURE 3:**
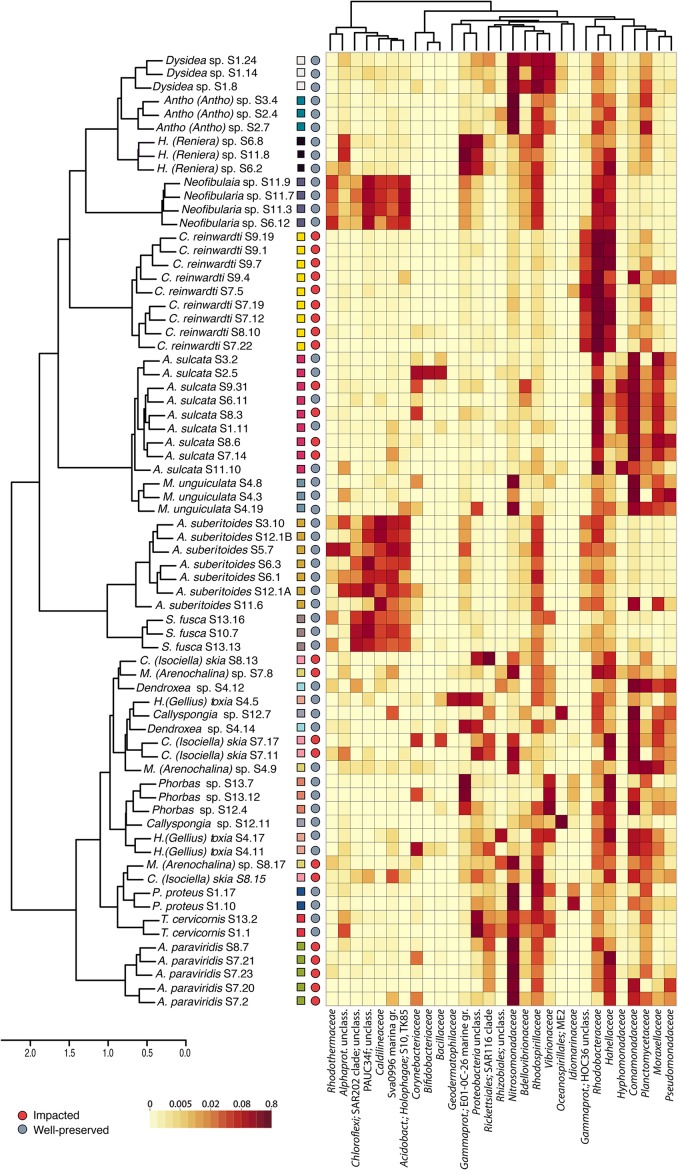
Heatmap of the sponge bacterial composition at the family level. Only taxa with relative abundances higher than 1% in any of the samples are shown, with abundance represented in the color temperature bar. Sponge samples (*y*-axis) and bacterial taxa (*x*-axis) are organized according to a hierarchical clustering based on Bray-Curtis dissimilarity matrices (at the level of ZOTU and family, respectively). Square colors correspond to different sponge species, and circle colors indicate the site of collection: impacted (red) or well-preserved (gray).

We obtained a total of 15,712 high-quality ZOTUs (Zero-radius Operational Taxonomic Unit, 100% identity) corresponding to 48 bacterial phyla, with *Proteobacteria* (52.9%)*, Actinobacteria* (2.8%), *Acidobacteria* (2.4%), *Chloroflexi* (2%), and *Planctomycetes* (1.7%) being the most abundant taxa. Bacterial communities of HMA sponges (*Neofibularia* sp., **A. suberitoides**, and *Suberea fusca* clearly differed from those of LMA sponges (Figure 3). *Chloroflexi*, PAUC34f, *Caldilineaceae* and Sva0996 marine group were consistently associated to the HMA sponges.

The cluster analysis showed that the sponge-associated bacterial communities were closely related to their host-species regardless of the environment (impacted vs. well-preserved) where the sponges were living, with, in general, low variation among replicates of a species (Figure 3). Indeed, we found a strong effect of host identity on the composition of the sponge microbiomes (*adonis*: Pseudo-*F*: 4.46, *R*^2^ = 0.593, *p* < 0.001), but a negligible effect of the environment (*adonis*: Pseudo-*F*: 1.07, *R*^2^ = 0.008, *p* > 0.05). Thus, each sponge species presented a unique microbiome that differed from that of other sponges and from that of the surrounding seawater (Supplementary Figure S4), even if they were living in the same environment (Figure 3). Planktonic communities showed significant differences in bacterial composition between types of habitats (*adonis*: Pseudo-*F*: 2.2, *R*^2^ = 0.23, *p* < 0.05, Supplementary Figure S4A). Shannon diversity was higher for the seawater bacterial communities in the well-preserved habitat than in the impacted habitat, but differences were only statistically significant at an alpha value of 0.07 (*Kruskal–Wallis* test: *p*-value = 0.07), likely due to the unbalanced design (3 vs. 6 replicates) and the large variation in the Shannon diversity Index across replicates at the polluted environment (Supplementary Figure S4B).

We looked in further detail into the bacterial composition of the two purportedly opportunistic species that dominate the perturbed (polluted) environments (*C. reinwardti* and *Amphimedon paraviridis*). A common feature of these two species was that their bacterial communities were dominated by a few taxa such as *Candidatus Branchiomonas* (42.85 ± 12.5%) and *Endozoicomonas* (12.74 ± 12.1%) in *A. paraviridis*, and members of the family *Rhodobacteraceae* (58.54 ± 22%) and *Endozoicomonas* (21.63 ± 12.9%) in *C. reinwardti* (Figure 3). In both sponges, only two bacterial taxa achieved >60% of the whole microbiome. Moreover, these two taxa were consistently found across all species replicates (i.e., they belong to the sponge core). A single *C. Branchiomonas* ZOTU made up 56% of the total core reads of *A. paraviridis* and 6 ZOTUs belonging to *Rhodobacteraceae* family made up 64% of the total core reads in *C. reinwardti.* Moreover, both species had in their core communities ZOTUs belonging to *Endozoicomonas* and *Shewanella* at abundances higher than 10 and 1%, respectively. Indval analysis (Supplementary Figure S5) showed that some bacteria were significantly associated to the impacted environment (Indval > 0.7, *p*-value < 0.01) but only *Rhodobacteraceae* and *Shewanella* were found at high abundances in the sponges of that habitat.

### Between Environment Comparisons: General Microbiome Ecological Descriptors

To test for microbiome differences among replicates of the same species living in the two environments, we focused on the unique species equally found in both habitats: *Amphimedon sulcata.* No influence of the environment could be detected for inter-individual variation (PERMANOVA: *R*^2^ = 0.128, *p* > 0.05) and no differences (*t-*test*, p*-value > 0.05) were detected for intra-species dispersion, core size and Shannon diversity of the *A. sulcata* bacterial communities between individuals from both environments.

**FIGURE 4:**
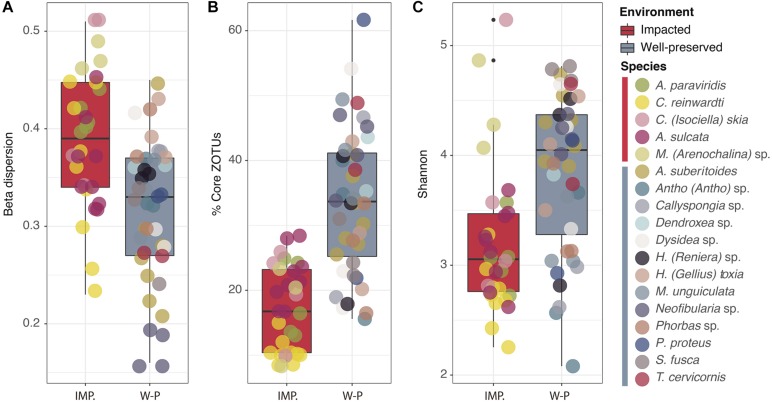
Box plots comparing the intra-species dispersion **(A)**, core size **(B)** and Shannon diversity **(C)** of the sponge microbiomes between impacted (red) and well-preserved (gray) environments. Replicates of the same species are depicted in the same dot color.

Intra-species dispersion of the sponge microbiomes was species-specific in all the species tested, with *Neofibularia* sp., *Thrinacophora cervicornis*, and *A. suberitoides* having the lowest dispersions and *Mycale (Arenochalina)* sp., *Clathria* (*Isociella*) *skia*, and *A. paraviridis* having the highest (Supplementary Figure S6A). Interestingly, intra-species dispersions were higher in impacted than in well-preserved environments (*t*-test: *p*-value < 0.001; Figure 4A). That is, the bacterial communities of sponge replicates from well-preserved environments are more similar to each other than seen for those sponge replicates from impacted environments. Consequently, intra-species dispersion was negatively correlated to the size of the sponge bacterial core (*R*_*p*_ = −0.65, *p*-value < 0.01: Supplementary Figure S7): the higher the intra-species dispersion, the lower the size of the core and the larger the variable bacterial community of the sponge species. *C. reinwardti, Mycale* (*Arenochalina*) sp. and *A. paraviridis* had the smallest core sizes (10.55, 17.34 and 18.44%, respectively), whereas *T. cervicornis, Protosuberites proteus, Monanchora unguiculata*, and *Neofibularia* sp. (43.71, 41.78, 41.73, and 41.72%, respectively) had the largest ones (Supplementary Figure S6B). Overall, the bacterial core sizes of the sponge species living in the impacted environments were lower (*Kruskal–Wallis* test: *p*-value < 0.001) than those of the sponges living in the well-preserved environments (Figure 4B). Shannon diversity indices (H′) of the sponge microbiomes were also species-specific and ranged from 2.7 to 4.7 (mean values for *C. reinwardti* and *S. fusca*, respectively) (Supplementary Figure S6C), which were lower in impacted than in well-preserved environments (*Kruskal–Wallis* test: *p*-value < 0.001) (Figure 4C).

**FIGURE 5:**
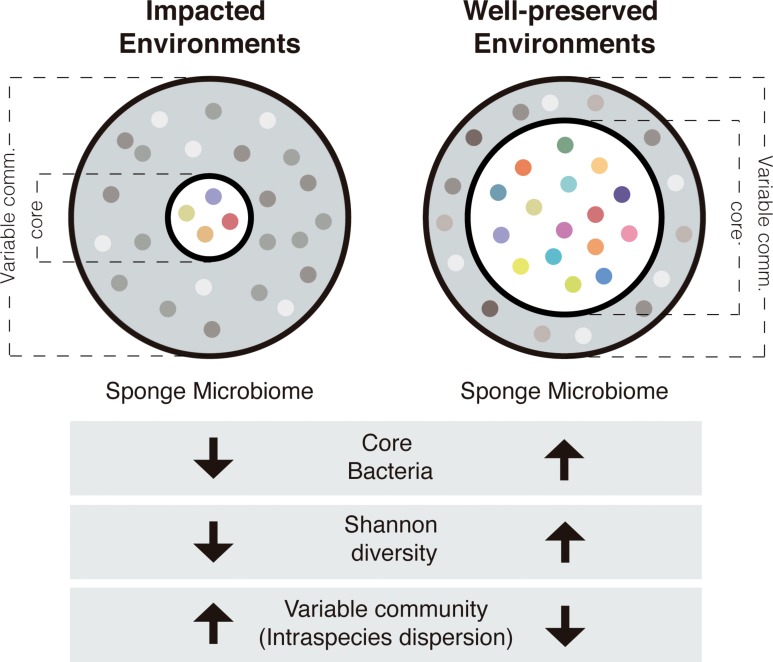
Schematic representation of the sponge microbiomes in impacted and well-preserved environments. Gray fraction represents the variable community and white fraction represents the core community. Each colored dot corresponds to a single bacterial species.

To sum up, microbiomes of sponge species living in the impacted environment had in general, higher intra-species dispersion, lower core size and lower bacterial diversity, than the microbiomes of sponges living in the well-preserved environments (Figure 5).

## Discussion

### Sponge Ecology: Well-Preserved vs. Impacted Environments

We have found clear differences between the sponge assemblages from well-preserved and impacted environments of Nha Trang Bay, with different species, lower sponge richness and diversity but higher sponge density (individuals/m.) in the perturbed zones. Similar decrease in species richness (S) and Shannon diversity index (H′) due to anthropogenic impacts has been previously reported for the coral communities in the study area (Tkachenko et al., 2016) and for sponge diversity in different oceans (Powell et al., 2014; Easson et al., 2015). Indeed, low diversity but high abundance with dominance of a few species is a common feature of many polluted habitats (Piola and Johnston, 2008; Powell et al., 2014). However, although our sampling captured differences in diversity between habitats quite acceptably, rarefaction curves for accumulated species predict an insufficient sampling effort to completely catch the true species richness.

In the Nha Trang region, marine cultures are producing chronic eutrophication and high sedimentation rates in some areas. As expected, the composition of sponge communities severely changes in those perturbed areas, with only a few sponge species inhabiting there. Body architecture and physiological traits, such as sediment removing mechanisms through mucus production (Bell et al., 2015; Schönberg, 2015, 2016) have been reported and allow sponges to survive under anomalous sedimentation rates (Pineda et al., 2017; Strehlow et al., 2017), as those resulting from severe eutrophication (Ralph et al., 2006). Some of these mechanisms are indeed displayed by the most abundant sponge species inhabiting the polluted habitat of Nha Trang (e.g., *C. reinwardti*, *A. paraviridis*, *A. sulcata*), as the sponge surfaces appear completely clean whereas a dense layer of sediment covers the remaining substrate (Figure 2). However, a series of xenobiotic compounds, resulting from an intense mariculture, are also released to the water at the impacted study area (Nguyen et al., 2016), so that biological/ecological sponge traits other than resistance to sedimentation, are expected to contribute to the observed abundance of a few species in those areas.

### Sponge Microbiomes: Shannon Diversity, Intra-Species Dispersion and Core Community

The bacterial communities of the study sponges show a high fidelity to the sponge species independently of the environmental conditions where the host species are living, as reported for many other sponge species (Luter et al., 2012, 2014; Simister et al., 2012; Bosch and Miller, 2016; Webster and Thomas, 2016; Gantt et al., 2017; Glasl et al., 2018).

Experimental studies repeatedly show a high resilience of the sponge microbiota in species exposed to a range of environmental variables, such as irradiance, temperature, salinity, acidification, and contrasting habitats (Erwin et al., 2012; Pita et al., 2013a, b; Cárdenas et al., 2014; Luter et al., 2014; Ribes et al., 2016; Glasl et al., 2017). A similar absence of effects on microbiome composition, and diversity was reported for the sponge *Gelliodes obtusa* under eutrophic conditions from mariculture (Baquiran and Conaco, 2018). As in the mentioned study, similar microbiomes were found in our study sponge *A. sulcata*, inhabiting either well-preserved or polluted environments. Thus, some haplosclerid sponges seem to tolerate eutrophication pressures although they can also inhabit well-preserved habitats, suggesting that they are sponges able to grow in a broad range of environmental conditions.

Most of the above-mentioned experimental studies focus on ecological adaptation of the sponge microbiomes to the assayed conditions but instead they found microbiome stability across treatments. Although surprising at first sight, microbiome resilience is indeed logical if we consider the concept of hologenome evolution (Rosenberg and Zilber-Rosenberg, 2018) and that these microbial-eukaryote symbioses have been evolutionarily fixed thousands of years ago (Sirová et al., 2018). Seemingly, structural microbiome stability has also been reported for few coral species across environmental gradients, which suggests resilience of microbiomes to environmental fluctuations or stress also in corals (Sawall et al., 2014; Grottoli et al., 2018; Pogoreutz et al., 2018).

Sponge microbiomes are species-specific in both environments, with no differences between individuals of the same species (i.e., *A. sulcata*) living at both habitats. However, significant differences in community metrics are revealed when the microbiomes of the sponge assemblages, as a whole, were compared between environments, with some particular bacterial groups proliferating in the polluted sites. Thus, even though the conditions of the perturbed environment cannot modify the evolutionarily fixed microbial communities of the sponge species (i.e., microbiomes do not change as a result of ecological adaptation to environmental conditions in species living at both habitats), they might influence the ecological distribution of the sponge species.

The sponge microbiomes of the polluted sites show a significantly lower Shannon diversity than sponge microbiomes in well-preserved environments. This is in accordance with the agreed general loss of biodiversity in impacted environments (Rygg, 1985). However, community success does not seem to directly depend on its diversity, but on its ability to respond to particular environmental conditions (McCann, 2000; Evans et al., 2017, 2018; Glasl et al., 2018). Indeed, the few species proliferating in the polluted environments of Nha Trang Bay (i.e., *C. reinwardti* and *A. paraviridis)* show a high density and a large coverage in the area, regardless of their low diversity microbiomes.

Moreover, the microbiomes of the sponges inhabiting the polluted sites show a higher intra-species dispersion than those of the sponges from well-preserved environments. The high microbiome dispersion in the former might indicate that the sponges living in the polluted environments, despite being visibly healthy are subjected to some stress. The Anna Karenina principle (Zaneveld et al., 2017), which proposes that variability is higher in dysbiotic than in healthy individuals of the same species, might be extended to species assemblages, according to our results. That is, bacterial communities of sponges in general, would be more variable in impacted than in well-preserved reefs. Similar trends in alpha and beta diversity metrics to those found in these contrasting environments have been also observed in successional stages of bacterial communities from primary, supposedly more stressed, to late or more mature (Ortiz-Álvarez et al., 2018).

The bacterial cores (Astudillo-García et al., 2017), predicted to play crucial roles in the sponge functioning (Cárdenas et al., 2014; Pita et al., 2018), represent a low percentage of the total microbiome in the study sponges inhabiting the polluted site. Consequently, most bacteria in these species are transient and thus, may be site-variable. This is confirmed by a negative correlation between the core size and the intra-species dispersion (variable community) of sponge microbiomes. Conversely, sponges inhabiting the well-preserved study sites contain large diverse bacterial cores that might be difficult to maintain in perturbed habitats, as both, theory and empirical evidence point to the simplification of ecological communities in those habitats (Piola and Johnston, 2008). As for the seawater bacteria communities, Shannon diversity was lower and variation between replicates was higher at the polluted habitats than at the well-preserved environments. Thus, seawater bacteria communities follow a similar pattern as for among replicates variation and alpha diversity than sponge microbiomes at both environments.

Moreover, the simplified microbial systems of the perturbed study habitats contain certain bacterial species that might play a role in the holobiont success.

*Rhodobacteraceae* members and *C. Branchiomonas* are major members of core communities of the two dominant sponges in the polluted sites (*C. reinwardti* and *A. paraviridis*), pointing to a potential function of these bacteria in the sponge success by facilitating them to cope with some pollutants. The family *Rhodobacteraceae* includes key players in biogeochemical cycling (Simon et al., 2017) and several members with chemotrophic anaerobic metabolisms, which are able to oxidize the noxious hydrogen sulfide present in eutrophic environments (Zhang et al., 2013). Members of the *Nitrosomonadaceae* family (i.e., *C. Branchiomonas*) are reported to be ammonia oxidizers (Prosser et al., 2014) and have been found to be dominant symbionts in sponges, suggesting that they may represent a source of bioavailable nitrogen for their hosts (Matcher et al., 2017). Also remarkable is the high abundance of *Endozoicomonas* in these sponge species. This genus, with various marine species distributed worldwide (Neave et al., 2016), is commonly found in close associations with sponges (Nishijima et al., 2013). Functions related to sponge health (Gardères et al., 2015), bromopyrrole production (Haber and Ilan, 2014), carbohydrate fermentation/nitrate reduction (Nishijima et al., 2013), and antibiotic production (Rua et al., 2014) in sponge hosts have been proposed (Neave et al., 2016). Thus, sponge-associated *Endozoicomona*s might play biological functions in the study sponges by participating in the nitrogen and sulfur cycles, influencing the inter-species interactions of the microbial community by producing antimicrobial compounds or signaling molecules, as reported for other sponges (Morrow et al., 2015). Finally, *Shewanella* is also found in the core of the two species at relatively high abundances at the impacted habitats. Members of this genus have been reported to reduce heavy metals, sulfates, nitrates, and chromates (Fredrickson et al., 2008), which are expected to be abundant in the polluted study habitats due to the antifouling paints containing heavy metals and residuals from industrial activities. The purported functions of the bacteria associated with the dominant sponges in the impacted habitats suggest a plausible role of the sponge microbiomes in detoxification of the seawater at a local scale. Indeed, sponges have been reported to exert a bioremediation effect (Milanese et al., 2003) in zones where they are abundant as they process high volumes of water per day (Leys et al., 2011), but further studies would be needed to confirm the real functionality of these bacteria in our study sponges.

## Conclusion

Overall, the microbiomes might play a certain role in determining the presence and proliferation of sponge species in polluted environments. Low core size, low diversity, and high intra-species dispersion are microbiome features shared by the study sponges inhabiting the prospected polluted environments, which suggests that less complex microbiomes are favored under degraded environmental conditions. Thus, the microbial communities associated with sponges mimic the reduction of diversity showed by animal or plant assemblages at the ecosystem scale.

Under the context of climate change, it has been proposed that coral reefs may change to sponge dominated reefs (Bell et al., 2013) due to the lower sensitivity of sponges to ocean acidification and eutrophication. However, few sponges are favored by these stressful conditions and, as shown in the present study, we may expect to face a scenario with less diverse but highly abundant sponge assemblages with low complexity microbiomes.

## Data Availability

Raw 16S rRNA gene amplicon sequences are available in the SRA archive under the project number PRJNA453898 (https://www.ncbi.nlm.nih.gov/sra/?term=PRJNA453898). Accession numbers for sponge eukaryotic sequences are reported in the author’s previous publication (Turon et al., 2018).

## Author Contributions

MT and MU conceived the study. MT, JC, and XT-M performed the analyses. MT and MU wrote the manuscript. JC, XT-M, and EC commented on later versions of the manuscript.

## Conflict of Interest Statement

The authors declare that the research was conducted in the absence of any commercial or financial relationships that could be construed as a potential conflict of interest.

## Footnotes

1 http://www.mrdnalab.com/
