# Supplementary material for: Sponges and Their Microbiomes Show Similar Community Metrics Across Impacted and Well-Preserved Reefs
Source: Front Microbiol. 2019 Aug 22;10:1961. doi: 10.3389/fmicb.2019.01961 (PMC6713927; doi:10.3389/fmicb.2019.01961)
Supplement: Supplementary file 2 [file Data_Sheet_2.PDF]

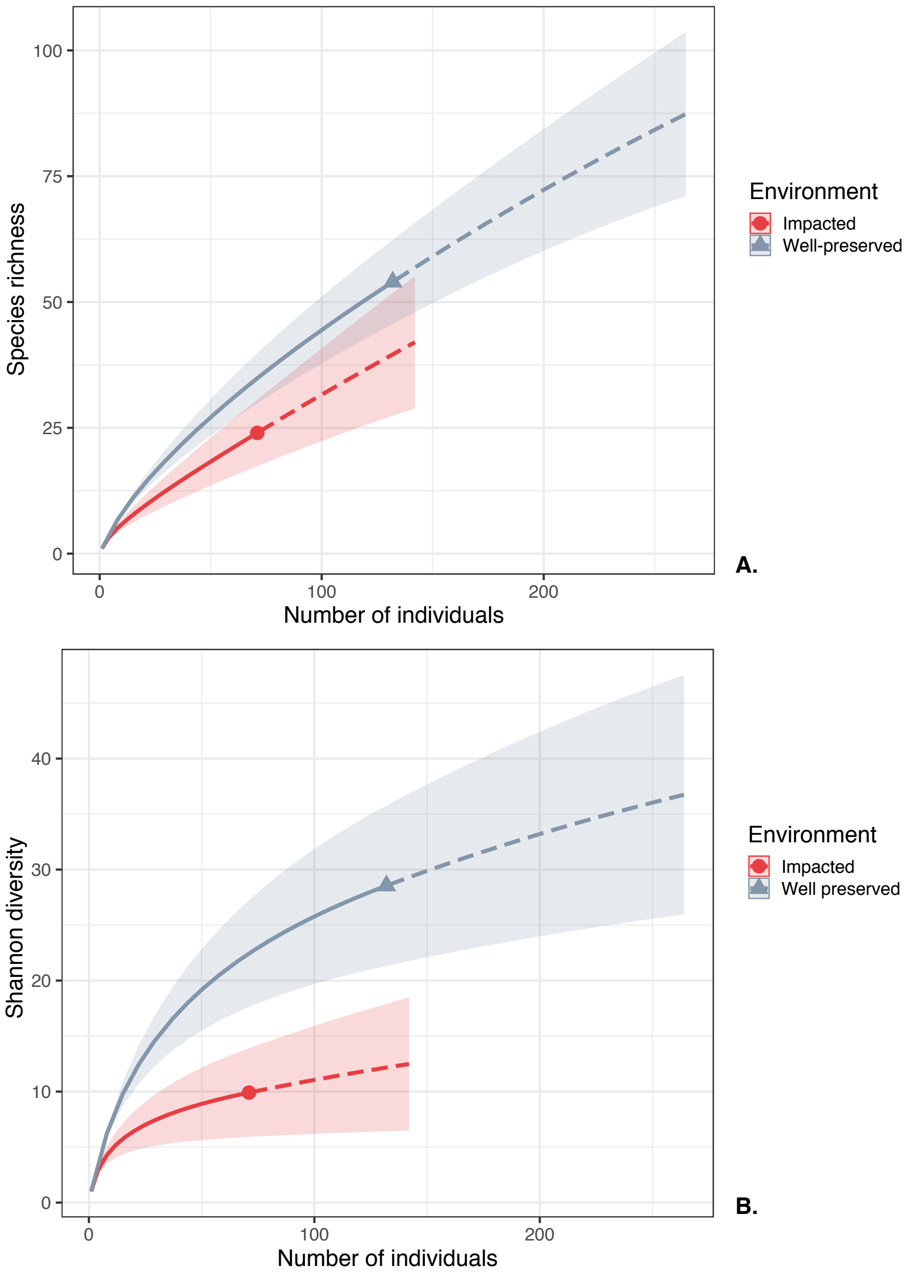

Figure S2 Sponge species richness (A) and Shannon diversity (B) for a given number of individuals in impacted (red) and well-preserved (grey) environments. Continuous lines represent interpolated values from the observed data and discountinuous lines represent extrapolated data.
