# Supplementary material for: Sponges and Their Microbiomes Show Similar Community Metrics Across Impacted and Well-Preserved Reefs
Source: Front Microbiol. 2019 Aug 22;10:1961. doi: 10.3389/fmicb.2019.01961 (PMC6713927; doi:10.3389/fmicb.2019.01961)
Supplement: Supplementary file 3 [file Data_Sheet_3.PDF]

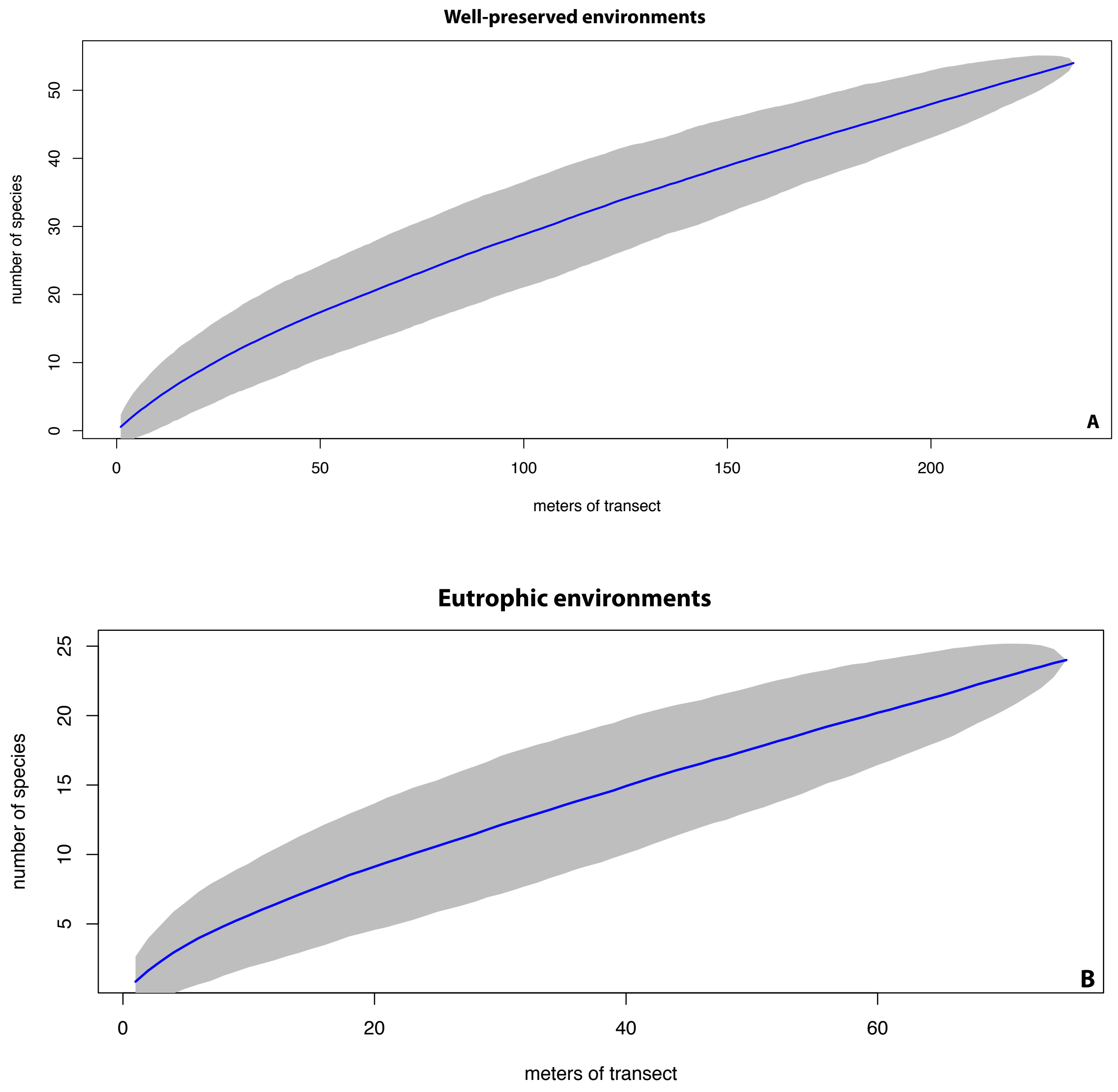

Figure S3 Species accumulation curves for well-preserved (A) and Impacted/Eutrophic sites (B). Blue line indicates the mean species accumulation at each sampled meter and grey area indicates the standard deviation.
