# Supplementary material for: Sponges and Their Microbiomes Show Similar Community Metrics Across Impacted and Well-Preserved Reefs
Source: Front Microbiol. 2019 Aug 22;10:1961. doi: 10.3389/fmicb.2019.01961 (PMC6713927; doi:10.3389/fmicb.2019.01961)
Supplement: Supplementary file 4 [file Data_Sheet_4.PDF]

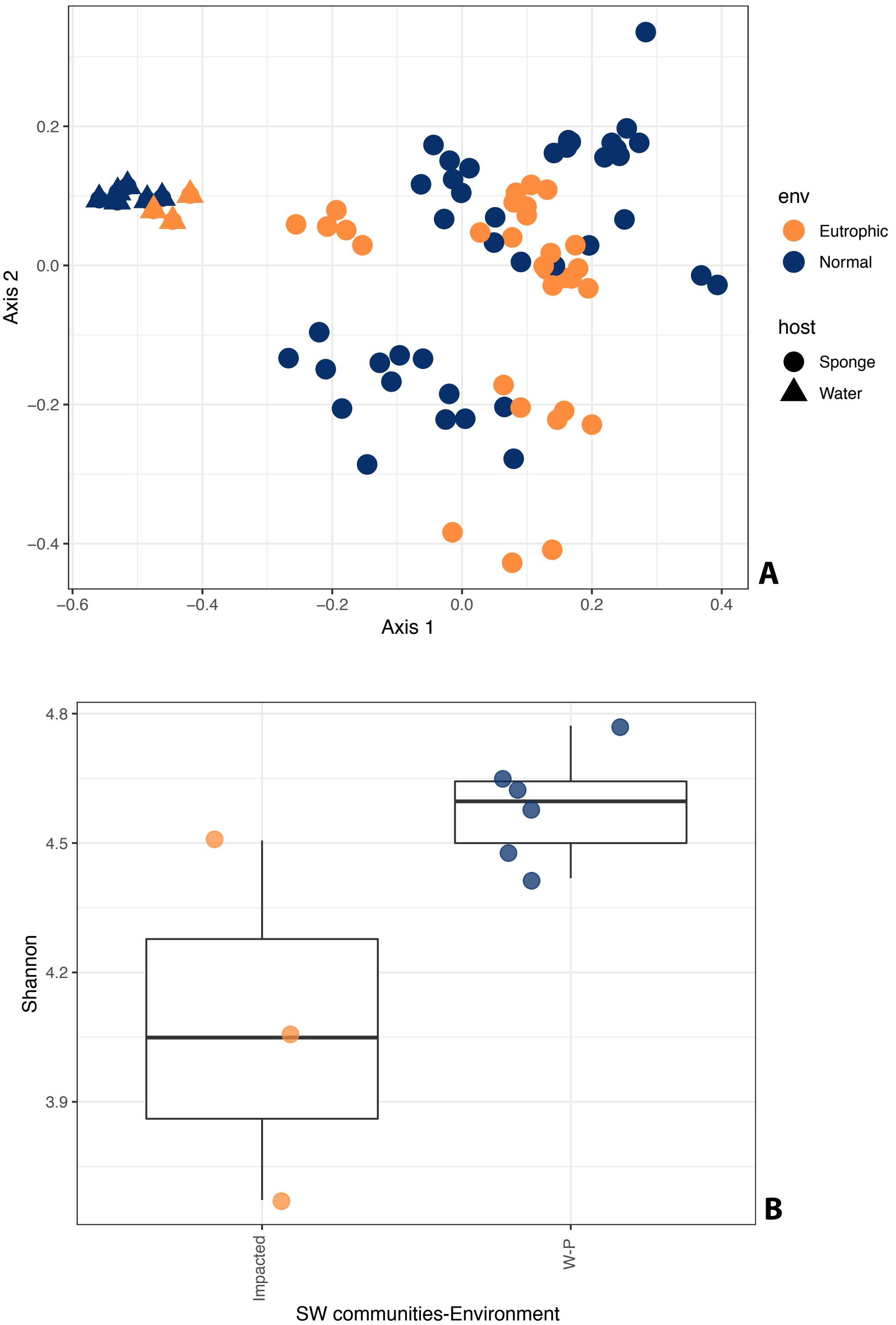

Figure S4. A) Non-metric multidimensional scaling (nMDS) ordination of the sponge bacterial communities ans sea-water bacterial samples based on Bray-Curtis distances coloured by their belonginess to impacted (orange) or well-preserved (blue) environment.
B) Box plot of the Shannon diversity indices of sea water bacterial communities from the impacted (orange) or well-preserved (blue) environments.
Water samples were collected during the same sampling dates as the study sponges (see Turon et al. (2018) for raw data).
