# Supplementary material for: Sponges and Their Microbiomes Show Similar Community Metrics Across Impacted and Well-Preserved Reefs
Source: Front Microbiol. 2019 Aug 22;10:1961. doi: 10.3389/fmicb.2019.01961 (PMC6713927; doi:10.3389/fmicb.2019.01961)
Supplement: Supplementary file 5 [file Data_Sheet_5.PDF]

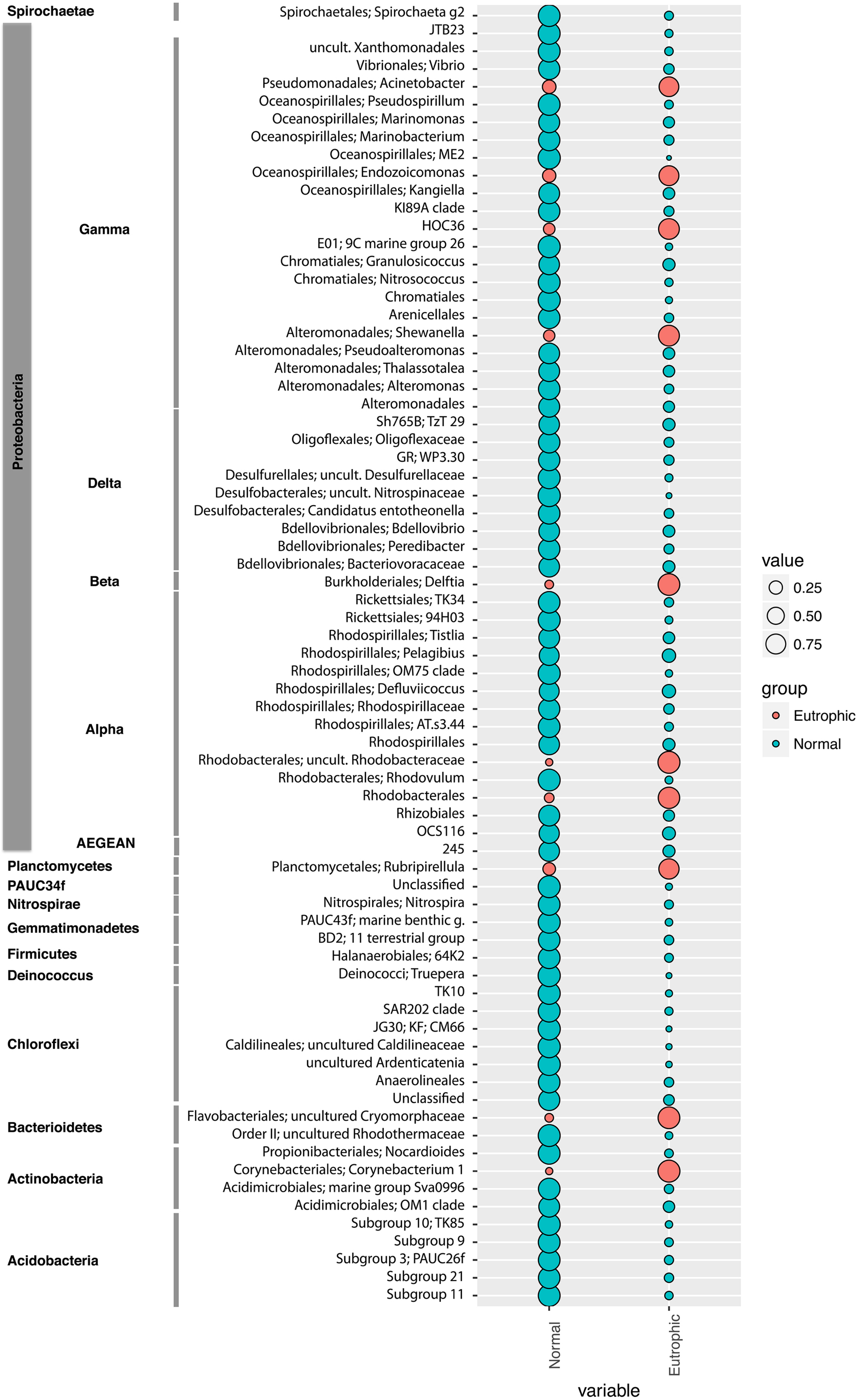

Figure S5 Results of the Indval analysis. Bacterial taxa represented were indicators of impacted (red) or well-preserved (green) environment (p-val < 0.01).
