# Supplementary material for: Sponges and Their Microbiomes Show Similar Community Metrics Across Impacted and Well-Preserved Reefs
Source: Front Microbiol. 2019 Aug 22;10:1961. doi: 10.3389/fmicb.2019.01961 (PMC6713927; doi:10.3389/fmicb.2019.01961)
Supplement: Supplementary file 6 [file Data_Sheet_6.PDF]

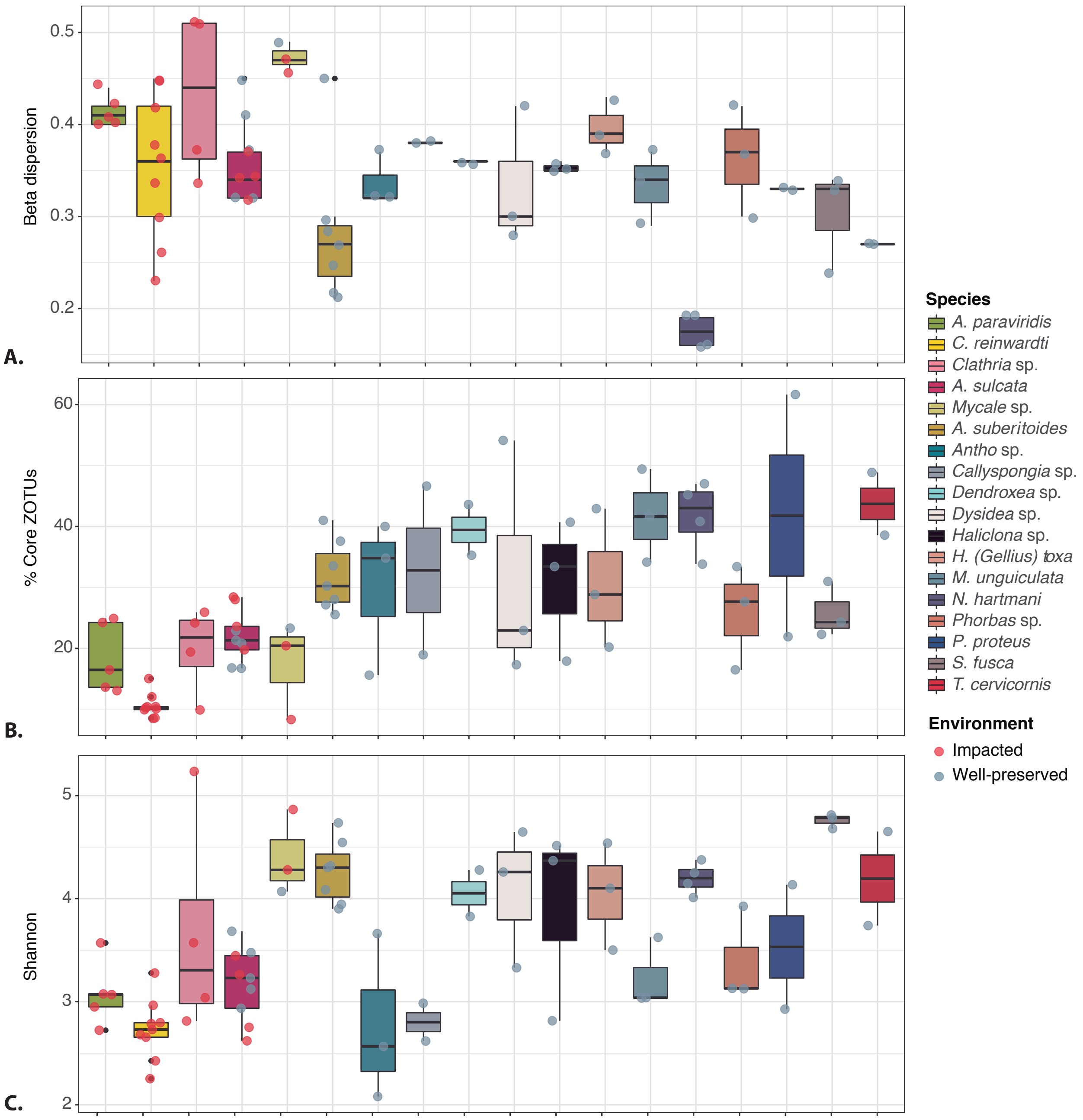

Figure S6 Box plots showing the intra-species dispersion (A), core size (B) and Shannon diversity (C) of each sponge species microbiome. Species replicates from impacted and well-preserved habitats are indicated by red and grey dots, respectively.
