# Supplementary material for: Sponges and Their Microbiomes Show Similar Community Metrics Across Impacted and Well-Preserved Reefs
Source: Front Microbiol. 2019 Aug 22;10:1961. doi: 10.3389/fmicb.2019.01961 (PMC6713927; doi:10.3389/fmicb.2019.01961)
Supplement: Supplementary file 7 [file Data_Sheet_7.PDF]

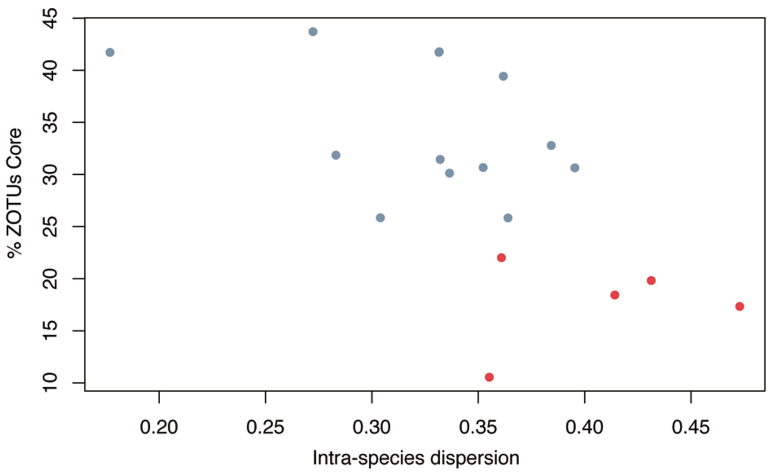

Figure S7 Relation between the intra-species dispersion and core size of the sponge microbiomes. Grey dots correspond to species from well-preserved habitats and red dots correspond to species from the impacted habitats.
